# Odor-Specific Daily Rhythms in the Olfactory Sensitivity and Behavior of *Aedes aegypti* Mosquitoes

**DOI:** 10.3390/insects9040147

**Published:** 2018-10-23

**Authors:** Diane F. Eilerts, Morgen VanderGiessen, Elizabeth A. Bose, Kyera Broxton, Clément Vinauger

**Affiliations:** Department of Biochemistry, Virginia Polytechnic Institute and State University, Blacksburg, VA 24061, USA; deilerts@vt.edu (D.F.E.); morgenvg@vt.edu (M.V.); eliza97@vt.edu (E.A.B.); kyerab4@vt.edu (K.B.)

**Keywords:** daily rhythms, biological rhythms, mosquitoes, vectors, olfaction, electrophysiology, behavior

## Abstract

Many biological processes and behaviors in mosquitoes display rhythmic patterns, allowing for fine tuning to cyclic environmental conditions. In mosquitoes, vector-host interactions are primarily mediated by olfactory signals. Previous studies have established that, in the malaria vector *Anopheles gambiae*, rhythmic expression of odorant binding proteins and takeout proteins in the antenna resulted in a corresponding rhythm in olfactory sensitivity to relevant host odors. However, it remained unclear how rhythms observed in olfactory sensitivity affect or explain rhythms in behavioral output, which ultimately impacts disease transmission. In order to address this knowledge gap, we quantified and compared patterns in locomotor activity, olfactory sensitivity, and olfactory behaviors in adult female *Aedes aegypti* mosquitoes. Here, we demonstrate an odorant-specific modulation of olfactory sensitivity in *Ae. aegypti*, decoupled from rhythms in olfactory behavior. Additionally, behavioral assays performed herein represent the first evidence of a time-dependence of the olfactory activation of behavior in *Ae. aegypti* mosquitoes. Results suggest that olfactory behavior of *Aedes* mosquitoes is modulated at both the peripheral (antenna) and central levels. As such, this work serves as a foundation for future studies aimed at further understanding the neural and molecular mechanisms underlying behavioral plasticity.

## 1. Introduction

Rhythmic biological processes are observed in a wide variety of organisms and optimize allocation of resources by coordinating behaviors and physiology with rhythmically fluctuating external conditions (e.g., cyclical changes in light, humidity, temperature, rhythmic activity patterns of prey and predators). Diel rhythms are directly driven by rhythmic environmental cues and differ from circadian rhythms in the sense that they are not controlled by endogenous biological clocks. Diel rhythms are therefore not sustained in the absence of rhythmic external cues. Together, diel and circadian rhythms enable organisms to adjust their physiology, metabolism, and behavior to predictable environmental cycles. 

Among disease vector insects, rhythmic patterns of locomotor activity [1,2,3,4], oviposition [5,6,7], blood-feeding [8], and olfactory behavior have been demonstrated in several species [9]. In most cases, they enable insects to synchronize their daily activity, and in particular their food-seeking behavior during periods when their hosts are resting, and are therefore the least able to defend themselves [10,11]. From a sensory perspective, these vector-host interactions are mediated to a large extent by olfactory signals. Volatile chemicals indeed serve as cues that allow these insects to detect, identify, and locate their hosts from distances at which they remain safe from the threat of defensive behaviors. Interestingly, and conversely to what had been suggested in other insects (e.g., [12,13,14]), disease vectors do not show a modulation in the overall sensitivity of their antennae for all chemical stimuli at the same time, but rather an activity and time-of-day dependent tuning of their olfactory sensitivity. For example, work on the malaria vector mosquito, *Anopheles gambiae*, showed that rhythms in antennal abundance of odorant binding proteins (OBP) lead to daily rhythms in olfactory sensitivity to major host-related chemicals, but not all rhythms are maintained in dark-treated mosquitoes [15]. In triatomine bugs, behavioral studies showed that the responsiveness to carbon dioxide is controlled by endogenous circadian clocks, while attraction to aggregation pheromones depends on environmental signals [16,17]. While these studies provide evidence that endogenous clocks and exogenous cycles act together to adaptively modulate sensory responses, whether rhythms in olfactory sensitivity are sufficient to fully explain the temporal modulation of the behavioral output remains unclear. 

The yellow fever mosquito, *Aedes aegypti*, is an ideal experimental model to explore the relationship between rhythms of olfactory sensitivity and temporal modulation of behavior as behavioral rhythms and bimodal activity patterns are well-documented, and biting behaviors are mainly observed during the second activity peak [1]. This additionally suggests a selective daily modulation of the olfactory behavior. However, the comparison in rhythmic sensitivity patterns between *Aedes* and *Anopheles* mosquitoes remains to be made. To close this knowledge gap, in the present study, we examined the relationship between daily variations in antennal sensitivity and behavioral responses in *Ae. aegypti* mosquitoes. Both plant- and host-related odors were tested during the peaks and troughs of their locomotor activity patterns. Finally, independently quantifying the flight activity patterns of the mosquitoes allowed us to decouple spontaneous activity from odor-evoked activation of flight.

## 2. Materials and Methods 

### 2.1. Insects

Two strains of *Ae. aegypti* mosquitoes were used for the experiments: Liverpool (actometer experiments; LVP-IB12, MR4, ATCC^®^, Manassas, VA, USA) and Rockefeller (all other experiments; MR-734, MR4, ATCC^®^, Manassas, VA, USA). Survey of the literature did not reveal any difference in the spontaneous locomotor activity of various strains of *Aedes aegypti* mosquitoes [1,18,19], discarding potential bias associated with strain differences. In addition, rhythms in peripheral sensitivity and olfactory behavior were analyzed within the same strain (Rockefeller) to prevent any bias being introduced. Larvae were raised in a 26 × 35 × 4 cm covered tray filled with ~1 cm of deionized water, at a density of approximately 200 larvae per tray. They were maintained under light:dark (LD) cycles of 12 h:12 h at 25 °C and 60 ± 10% humidity. Larvae were fed a daily diet of Hikari Tropic First Bites (Petco, San Diego, CA, USA). Pupae were isolated on the day of pupation and placed into mosquito breeding containers (BioQuip, Rancho Dominguez, CA, USA—1425, 1425DG). These containers were then transferred to opaque plastic containers (Rubbermaid Brute boxes, 71 × 44 × 38 cm) at least three days prior to experiments to allow mosquitoes to synchronize to a light cycle aligned with the experimenters’ work hours for one of the four tested *Zeitgeber* time (ZT) periods (0–2, 5–7, 10–12, 17–19) on a 12 h:12 h LD cycle where lights-on takes place at ZT 0 and lights-off at ZT 12. In all cases, mosquitoes tested at ZT 0–2 were taken from their rearing containers only after the onset of the lights, and all experiments conducted at ZT 17–19 were performed under red light illumination. For all experiments except actometer experiments, 6 ± 1 day-old adult female mosquitoes were used only once. 

### 2.2. Activity Analysis 

**Figure 1:**
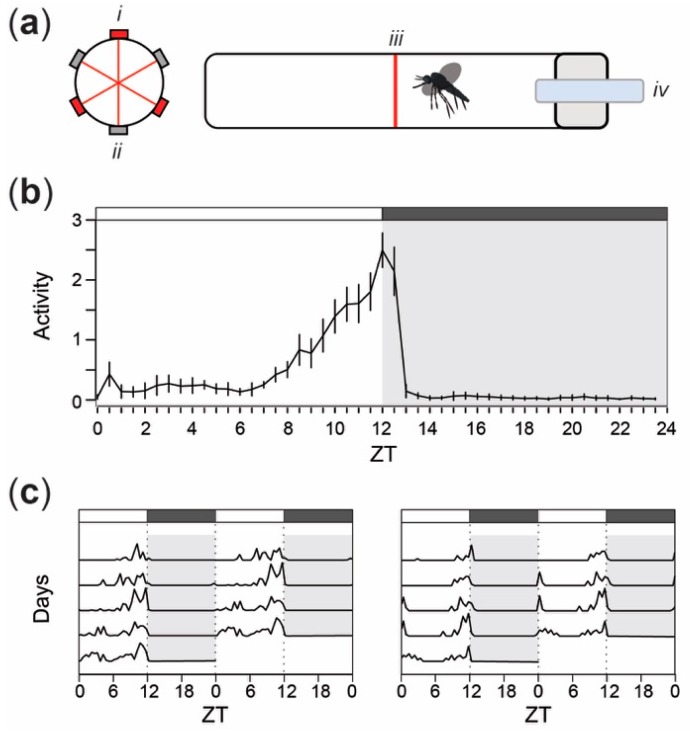
Locomotor and flight activity recordings in adult female *Ae. aegypti* mosquitoes show a bimodal pattern of activity, peaking in the last hours of the photophase. (**a**) Schematic representation of the locomotor activity monitoring system (Trikinetics LAM25, Waltham, MA, USA) showing a cylindrical glass tube containing an individual mosquito as viewed from the tube opening (left) and from the side (right). (*i*) Infrared laser emitters and (*ii*) detectors (*iii*) bisect each tube, which is enclosed with a (*iv*) sugar reservoir for constant access to 10% sucrose. (**b**) Mean locomotor activity pattern of 94 *Ae. aegypti* females in 12 h:12 h light-dark (LD) cycle. Values on the *x*-axis correspond to *Zeitgeber* time (ZT). Error bars represent the standard error around the mean. (**c**) Double-plotted actograms illustrating representative locomotor activity in two individual mosquitoes in 12 h:12 h LD cycles for 5 consecutive days, following a 48-hour period of light entrainment. Gray background and boxes above the actograms indicate the scotophase (i.e., dark period of the cycle) while white background and boxes depict the photophase (i.e., light-on phase).

Locomotor and flight activity of female mosquitoes were recorded using a locomotor activity monitor (Trikinetics LAM25, Waltham, MA, USA). The LAM25 system consists of a vertical printed circuit board (PCB) with 32 openings, each equipped with 3 sets of infrared emitters and detectors. One to two days after emergence, individual mosquitoes were placed in glass cylindrical tubes with access to 10% sucrose at one end (Figure 1a). Mosquitoes were maintained in the tubes for a duration of 8 days. The tubes were positioned through the openings on the panel so that the infrared beams were located at the approximate center of each tube. Daily locomotion was recorded as the number of beam crossing per 30 min intervals using the DAMSystem3 Software (Trikinetics, Waltham, MA, USA). All recordings occurred in a light-proof enclosure with its own lighting system, which consisted of a light-emitting diode (LED) light (800 Lumen, Philips, Amsterdam, The Netherlands) timed to a 12 h:12 h LD cycle. Data analyses were performed on locomotor activity for a total of 94 mosquitoes and over either 4 or 5 consecutive days starting on the third instance of ZT 0 (i.e., on the third day), in order to ensure mosquitoes were appropriately entrained to the LD cycle. Individuals that were found dead at the end of the experiments were discarded from calculations after the last timepoint being recorded as active.

### 2.3. Olfactory Sensitivity

**Figure 2:**
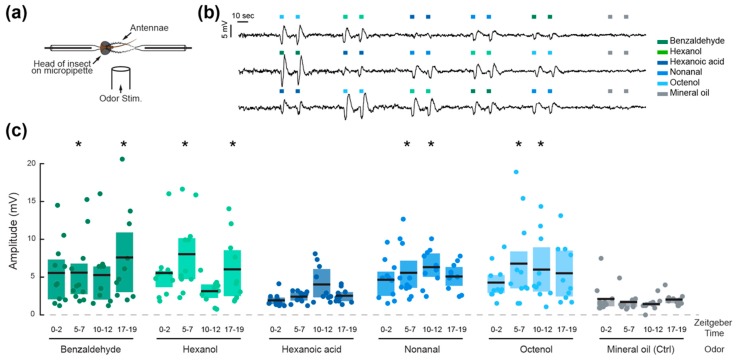
Olfactory sensitivity is time and odorant specific in adult *Ae. aegypti* females. (**a**) Schematic of the electrophysiological preparation for electroantennogram recordings. (**b**) Three representative traces depicting typical voltage deflection after olfactory stimulation of two consecutive pulses of five odorants, delivered in a randomized order. (**c**) Mean amplitude of the electrophysiological responses to the five odorants and mineral oil control at four different times of day. Each dot represents the response of an individual mosquito, black lines indicate the mean response at each time point, and shaded areas represent the first and last quartiles. Asterisks denote responses that are significantly different from the respective mineral oil control (Tukey post-hoc test, *p* < 0.05).

Electroantennography was used to quantify the olfactory sensitivity of 10–12 mosquitoes tested at four different times (ZT 0–2, 5–7, 10–12, and 17–19), corresponding to dawn, midday, dusk, and midnight for the insects. Mosquitoes tested at ZT 17–19 were prepared in functional darkness under red light, not visible by the mosquitoes [20], and tested in dark conditions. Individuals tested were immobilized on ice before their head was excised with surgical micro-scissors and mounted on an indifferent (i.e., reference) glass electrode. The recording electrode accommodated the two antennae of the excised head after the tips of the antennae were clipped to provide a better contact (Figure 2a). Both electrodes consisted of oxidized silver wires inserted into drawn-out glass capillary tubes filled with saline solution [21]. The signal from the electrodes was amplified by a microelectrode AC amplifier (Model 1800; A-M Systems, Sequim, WA, USA) and was filtered with a HumBug noise eliminator (Quest Scientific, North Vancouver, BC, Canada). The signal was then recorded using a winEDR Strathclyde Electrophysiology Data Recorder V3.8.0 (University of Strathclyde, Glasgow, UK).

Volatiles tested were benzaldehyde, nonanal, hexanol, hexanoic acid, and 1-octen-3-ol (octenol). Chemicals were obtained from Sigma-Aldrich and were prepared by dilution (1:100) in mineral oil (M8410-1L) for final concentrations of: 98 mM benzaldehyde (≥99.5%, 418099-100ML); 58 mM nonanal (95%, N30803-25G); 80 mM hexanol (Anhydrous, ≥99%, 471402-100ML); 80 mM hexanoic acid (≥99%, 153745-100G); and 65mM octenol (98%, O5284-25G). The rationale behind this choice was to test plant- (benzaldehyde and hexanol) and host-emitted compounds (hexanoic acid, octenol), at concentrations that are not limiting factors for quantifying mosquitoes’ olfactory sensitivity. It is worth noting that nonanal can be found in the headspace of both plants and animals [22,23]. Pure mineral oil was used as a control. The stimuli were delivered by pipetting 40 microliters of each odor onto a piece of Whatman filter paper (GE Healthcare Bio-Sciences, Pittsburgh, PA, USA) in a respective glass syringe. The tip of the syringe was then inserted into a glass tube delivering a constant air flow (30 cm·s^−1^) from a medical air tank (Praxair, Inc., Danbury, CT, USA), the end opening of which was 12.3 mm in diameter and positioned about 5 mm from the mosquito head. The tip of the syringe was positioned 9.12 cm upstream of the opening. For each odor, two 2-second long pulses were delivered (2.3 cm·s^−1^) with a 10 second inter-pulse interval. In other words, the odorant concentrations were therefore further diluted ~13 times. Odor pulses were triggered by a 3-way solenoid valve (The Lee Company, Westbrook, CT, USA) controlled by a custom-written Matlab script (The MathWorks Inc., Natick, MA, USA). All five odors were delivered in a series to each mosquito head, and the order was randomized in Matlab. Mineral oil was used as a control and delivered before and after the five odors. Preparations that displayed background levels below one standard deviation above the mean background level of our sampled population (indicating proper electrophysiological montage) and for which no significant responses (i.e., not different from responses to mineral oil) were observed, were considered as non-responsive and discarded from the analysis. For each time point, 10–11 responsive mosquitoes were used for the analysis.

### 2.4. Olfactory Behavior 

**Figure 3:**
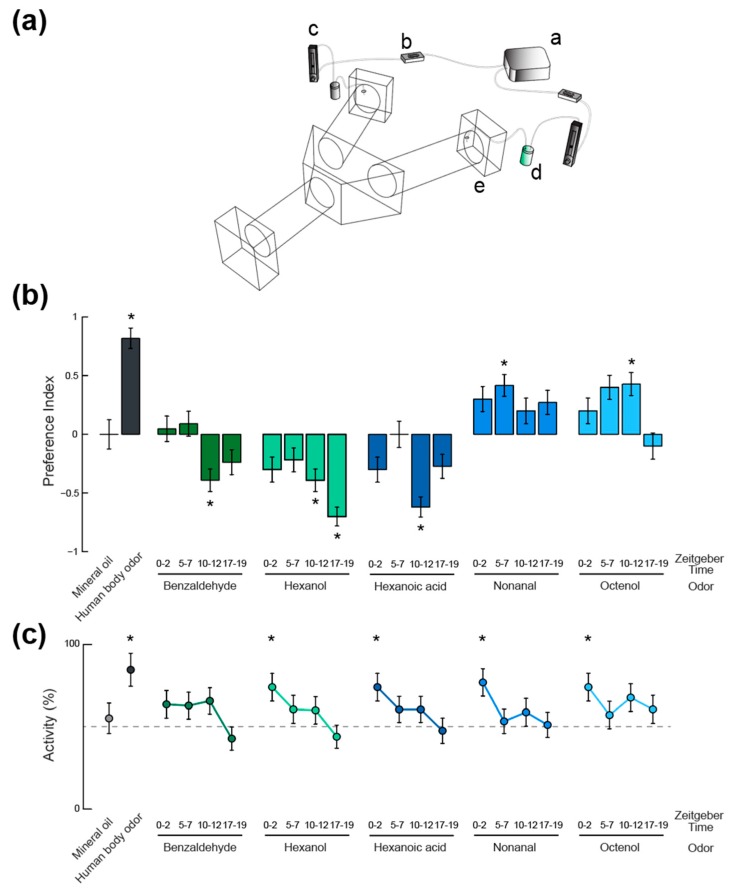
Time-of-day and odorant-specific olfactory activation and behavioral responses of *Ae. aegypti* adult female mosquitoes. (**a**) Schematic representation of the Y-maze olfactometer used in behavioral assays. (*i*) air pump; (*ii*) charcoal filter; (*iii*) flowmeter; (*iv*) scintillation vial containing either the odorant stimulus or the solvent only; (*v*) delivery of the odor laden air into the choice arms of the olfactometer. Mosquitoes are released into the entrance arm and fly upwind where they are forced to make a decision between the two choice arms, each delivering a different odor stimulus. (**b**) Mosquito odor preference represented as a preference index. Asterisks denote behavioral responses that were significantly different from chance (Binomial Exact test, *p* < 0.05). (**c**) Proportion of activity (i.e., proportion of mosquitoes making an active choice between the two choice arms of the olfactometer) under different olfactory contexts and at four different times of day. Asterisks denote proportions that were significantly different from activity levels in absence of odor (55%; Binomial Exact test, *p* < 0.05). In (**b**,**c**), error bars represent the standard error to the mean.

An enclosed Y-maze olfactometer [24] was used to quantify the behavioral responses of mosquitoes to the same plant- and host-related chemical compounds used in the electrophysiology experiments, at the same concentrations (i.e., benzaldehyde, nonanal, hexanol, hexanoic acid, and octenol). The olfactometer consisted of custom-cut acrylic with two choice arms and one entrance arm connected to a central chamber (100 cm long, 10 cm internal diameter, choice arms positioned at a 120° angle, Figure 3a). A constant airflow was generated by two fans (Rosewill, Los Angeles, CA, USA, air speed ~30 cm·s^−1^) mounted at the end of the choice arms and was filtered through a series of activated charcoal filters and honeycomb mesh (10 cm long) to create a contaminant-free laminar airflow. The olfactory stimuli were delivered through divided circuits of polytetrafluoroethylene (PTFE) tubing (McMaster-Carr, Elmhurst, IL, USA) and charcoal-filtered (~3 cm·s^−1^), further diluting the odorant concentrations 10 times. The air was then passed through scintillation vials containing either the tested odor or the control solution (mineral oil). Each respective line was attached to the distal end of the corresponding choice arm through small holes in each of the arms of the Y-maze. To avoid environmental bias, the position of the stimulus and control currents were randomly exchanged in the olfactometer arms between experiments. Statistical analysis did not reveal any preference for the left or right side of the olfactometer (n = 16; Binomial Exact test: *p* = 0.59) when mosquitoes were tested against two clean (i.e., mineral oil laden) air currents at ZT 10–12. All experiments were performed in a well-ventilated chamber at a constant temperature (26 ± 2°C) and relative humidity (50–70%). For each odor and time point, 16 < n < 23 mosquitoes made a choice and were taken into account for preference analysis.

### 2.5. Data Analysis

Activity data generated with the actometer, electrophysiological data, and binary data collected in the olfactometer were analyzed using R software (version 3.4.2 [25]). Activity data was analyzed by calculating the William’s Mean (Mw), a modified geometric mean to accommodate datasets with zero values [26], of the activity for all mosquitoes monitored. Activity data for individuals observed dead at the end of the experiment were excluded following the last recorded time of activity. A strong “startle response” in the activity was observed at the lights-off, similarly to observations made by others [18,27]. Activity data recorded at this time were excluded and instead interpolated by calculating the arithmetic mean between activity values recorded immediately before and after lights-off [18]. This was done in an effort to avoid representing artificially heightened activity due to the abrupt lighting change at lights-off. Instead, this exclusion represents the locomotor activity at this time in a manner that is more likely representative of *Ae. aegypti* activity in nature occurring at sunset. The cyclicity of the activity of individual mosquitoes was tested by means of Fisher’s Exact G-test, based on the null hypothesis of Gaussian white noise against the alternative of an added periodic component of unspecified frequency [28].

Trigger-averaged electrophysiological data were analyzed for each odor stimulus and time point. The amplitude of the odor-induced voltage deflection were extracted and used for the analysis (ANOVA and Tukey multiple comparisons of means). Binary data obtained in the olfactometer were compared to chance by means of the Binomial Exact test (α = 0.05). 

## 3. Results

The overall pattern of locomotor activity of female *Ae. aegypti* under a 12 h:12 h LD cycle was consistent with previously published observations (e.g., [1,18]). The mean daily activity of 94 individuals shows a bimodal diurnal pattern of locomotor activity (Figure 1b). A first slight increase in average activity levels was observed within the first two hours after lights-on, followed by a period of low activity until approximately four hours prior to lights-off when a second increase in activity occurred. Locomotor activity reached a peak at approximately ZT 12 and immediately decreased dramatically within the first hour after lights-off. During the scotophase (i.e., the period of darkness) that follows (i.e., ZT 13–24), we observed minimal levels of activity. Of the 94 tested females, 71.3% displayed significantly rhythmic activity patterns (Fisher’s Exact *G*-test, *p* < 0.05), similar to the two representative individual actograms depicted in Figure 1c. 

The peaks and troughs of locomotor activity were selected as *Zeitgeber* Times (ZT) for testing the peripheral olfactory sensitivity of the mosquitoes. The responses to five odorant volatiles (two aldehydes, two alcohols, and a carboxylic acid), were therefore tested at ZT 0–2, 5–7, 10–12, and 17–19 (Figure 2). The amplitudes of the odor-evoked responses were significantly influenced by the odorant stimuli (ANOVA; *F*_6, 557_, *p* < 0.001), the time of day (*F*_3, 557_, *p* = 0.044) and the interaction between the odorant and time of day (*F*_18, 557_, *p* < 0.001; Table 1). All odorants except hexanoic acid elicited significant electrophysiological responses compared to the mineral oil control pulses (Tukey post-hoc test; *p* < 0.05), but the patterns of daily olfactory sensitivity were odorant specific. Responses to octenol, nonanal, and hexanoic acid did not vary significantly throughout the day, while responses to benzaldehyde and hexanol significantly varied as a function of time (pairwise comparisons, Tukey post-hoc test, *p* < 0.05). The mosquitoes were most sensitive to benzaldehyde during the 17–19 h, to hexanoic acid during the 10–12 h, and to hexanol during the 5–7 and 17–19 h time periods. Interestingly, the proportion of responsive preparations (i.e., preparations that displayed at least one response significantly higher than baseline responses to mineral oil) followed the same trend as the locomotor activity rhythm, with 91.6 ± 4.8% of preparations responsive during the photophase, and 58.8% of preparations responsive during the scotophase (n = 35 and n = 17 respectively). The non-responsive individuals also displayed larger background noise levels. 

In order to test how these daily variations in olfactory sensitivity affect the behavioral responses of the mosquitoes, a Y-maze olfactometer was used to examine their olfactory behavior (Figure 3b). A neutral control, for which a choice between two clean air currents was given, showed no bias in the experimental set up as mosquitoes oriented randomly between the two arms (n = 16; Binomial Exact test, *p* > 0.05). A positive control, using human feet odor extract, confirmed the suitability of the olfactometer to reveal oriented behaviors (n = 11; 90.9% attraction, Binomial Exact test: *p* = 0.011). The host-emitted aldehyde nonanal, for which no peripheral modulation of the olfactory sensitivity was observed, elicited low levels of attraction compared to our positive control. Although no significant difference was observed between responses at different time points, only the ZT 5–7 tests induced a response that was significantly different from chance (i.e., a significant attraction; n = 24; 70.8% attraction, Binomial Exact test: *p* = 0.019). The other compound that did not show any response variation at the periphery, octenol, did elicit significantly different levels of behavioral response throughout the day. Only the responses to octenol at ZT 5–7 and 10–12 were either significantly different from chance or marginally significant (n = 20 and n = 21; 70% and 71.4% attraction; Binomial Exact test: *p* = 0.057 and *p* = 0.039, respectively). These levels of attraction were both significantly different from responses quantified during the scotophase (n = 20; Binomial Exact test: *p* = 0.021 and *p* = 0.013, respectively). For benzaldehyde and hexanol, the strongest behavioral responses were aversive (ZT 10–12, n = 23, 69.6% aversion, Binomial Exact test: *p* = 0.046 for benzaldehyde; ZT 10–12 and ZT 17–19, n = 23 and n = 20, 69.5% and 85% aversion, Binomial Exact test: *p* = 0.046 and *p* = 0.001, respectively, for hexanol) and were not observed in time windows during which the olfactory sensitivity was maximal for these chemicals (i.e., ZT 17–19 for benzaldehyde and ZT 5–7 for hexanol). Finally, responses to hexanoic acid were strongest during the maximum sensitivity window (ZT 10–12, n = 21, 81.0% aversion, Binomial Exact test: *p* = 0.004). Interestingly, all the other time windows showed responses that were not different from chance (20 < n < 23, Binomial Exact test: *p* > 0.05).

The activity levels in the olfactometer (i.e., the proportions of mosquitoes initiating flight and making a choice between one of the two decision arms), were consistent across odorant stimuli (Figure 3c). When compared to activity levels observed in the absence of odorant stimulus (55.1% of active mosquitoes; neutral control; N [i.e. n active mosquitoes + non-active individuals] = 29), the human feet extract (84.6% activity; Binomial Exact test: *p* = 0.027; N = 13), hexanol at ZT 0–2 (74.1% activity; Binomial Exact test: *p* = 0.033; N = 27), nonanal at ZT 0–2 (76.9% activity; Binomial Exact test: *p* = 0.018; N = 26), and octenol at ZT 0–2 (74% activity; Binomial Exact test: *p* = 0.034; N = 27) elicited significantly higher levels of flight initiations and decisions. Although not statistically significant, experiments conducted during mosquitoes’ night times (i.e., ZT 17–19) led to fewer mosquitoes making a choice in the olfactometer than for the neutral control (33 < N < 50; Binomial Exact test: *p* > 0.05). The lowest level of activity observed being in response to benzaldehyde at ZT 17–19 (42.8% of activity; Binomial Exact test: *p* = 0.059; N = 49).

## 4. Discussion

In this study, we investigated the interplay between locomotor activity, olfactory sensitivity, and olfactory behavior in the disease vector mosquito *Ae. aegypti*. Three distinct questions were addressed in the present work: at the peripheral (i.e., antennal) level, does the sensitivity to odorant volatiles vary throughout the day? How does this daily rhythm in sensitivity translate into behavioral outputs? Are behavioral responses to odors limited by locomotor activity rhythms?

In the first part of this work, we confirmed the locomotor activity patterns observed by others in previous studies [1,18], with a small activity peak in the first hours of the photophase and a larger peak in the last hours of the photophase. The locomotor activity is reduced between these peaks, both in the photophase and the scotophase. Therefore, the peak and trough time windows were selected for the rest of the study to provide a baseline for comparing olfactory sensitivity and behavior at equally spaced times. Our next step was to use electroantennogram (EAG) analysis to quantify the olfactory responses induced by host- and plant-derived odorant chemicals. We purposely used concentrations within the same order of magnitude and above concentrations that mosquitoes would experience in nature [29,30,31]. This allowed meaningful cross comparisons between odorants, and ensured that low responses observed in the electroantennogram recordings were not due to low concentrations, but actually indicated low sensitivity to that odor. In contrast to work conducted in the malaria vector *Anopheles gambiae*, in which daily patterns of olfactory sensitivity were conserved across most odorant chemicals [15], here we observed an odorant specific modulation of the sensitivity. The level of peripheral sensitivity has been hypothesized to be mostly driven by rhythms in the expression patterns of Odorant Binding Proteins (OBPs) and takeout proteins [15]. This hypothesis largely relies on the fact that carboxylic acids (e.g., hexanoic acid and lactic acid), which are hydrophilic host odor constituents and, therefore, do not require OBPs for detection, are detected similarly at different times of day in *An. gambiae* and *Culex pipiens* [15,32]. However, here we observed that, although not significant, sensitivity to hexanoic acid also seemed to be time-of-day dependent, suggesting that, in *Aedes* mosquitoes, other regulatory processes might be at play. 

Another particularity of *Ae. aegypti* mosquitoes is that, conversely to the nocturnal species mentioned above, these diurnal mosquitoes display a bimodal activity pattern (Figure 1; [1]). While in the presence of available human hosts, *Ae. aegypti* females were observed in equal numbers at both activity peaks in landing assays conducted in the field [33,34], other behaviors, such as the oviposition behavior, are limited to the second activity peak [35]. The odorant-specificity in sensitivity rhythms that we observed is therefore not surprising, as some chemical compounds might be used by the mosquitoes in specific behavioral and temporal contexts. Benzaldehyde and hexanol are plant-associated odors and it is understandable that sensitivity to these odors would peak in times that are not associated with host-feeding. Additionally, our behavioral results show that *Aedes* mosquitoes are more repelled by these odors in the late hours of the day (i.e., ZT 10–12 and ZT 17–19), implying that they are not likely to be sugar-feeding at these times, when they are often observed to be host-seeking. Alternatively, mosquitoes showed higher antennal sensitivity to host-related odors (in particular nonanal and octenol) in the middle and end of the photophase (ZT 5–7 and 10–12), which is in line with their peak host-seeking times during the day. Mosquitoes showed significant attraction to nonanal in the middle of the day (ZT 5–7) and octenol near the end of the day (ZT 10–12), further indicating that olfactory processes constrain mosquitoes to primarily be host-seeking as opposed to sugar-feeding at these times. The high sensitivity and behavioral responses to host odors at midday, when only low levels of spontaneous locomotor activity are otherwise observed, suggest that these anthropophilic mosquitoes are capable of being activated by host kairomones and respond to opportunistic host encounters.

Another example of odor-specific rhythm is provided by studies performed in triatomine bugs, which also display a bimodal activity pattern. They were observed to seek for a host to bite in the beginning of the night and for a refuge to hide at dawn [11]. More specifically, behavioral experiments showed that these insects respond to CO_2_ only during the first hours of the night and are attracted to refuge-associated aggregation pheromones only at sunrise [36]. Furthermore, analysis revealed that the circadian system is responsible for the modulation of the response to CO_2_, whereas the responsiveness to aggregation pheromones is driven directly by environmental factors [17]. Rhythmic pheromone detection has been extensively studied in other insects, such as cockroaches [37,38] and moths [39,40,41,42]. Further, the central circadian system has been established to modulate olfactory sensitivity in cockroach antennae [13,43] while moth antennae contain their own circadian pacemakers [44]. However, whether another level of modulation in the antennal lobes of mosquitoes may exist in these cases remains unknown. 

One key finding of the present study is that for some of the odorants tested, such as benzaldehyde and hexanol, sensitivity was maximal during resting periods. This seemingly paradoxical observation has also been made in other insects [12,13], and although various explanations have been proposed, the biological relevance of this phenomenon remains to be understood. Such behavioral rhythms in *Ae. aegypti* may be important for processes such as predator detection or opportunistic feeding or have another role in their complex and nuanced ecology. 

In this context, linking these peripheral rhythms in olfactory sensitivity with the behavioral response of the mosquitoes to the same odorants (tested at the same concentrations) appeared necessary. The first conclusion from our behavioral assays is that peaks in olfactory sensitivity do not necessarily align with peaks in behavioral response. For example, *Aedes* mosquitoes were not more repelled at night by benzaldehyde than they were at dusk, even though the electrophysiological responses were higher during the dark phase. Similarly, while EAG recordings showed maximal sensitivity to hexanol at ZT 5–7, no behavioral repulsion was evinced at this time of day. However, in some cases, such as in response to hexanol at night, both behavioral and antennal responses were high. Additionally, both olfactory sensitivity and behavioral repulsion to hexanoic acid were maximal at ZT 10–12. This suggests that the observed modulation of the behavioral output (i.e., the oriented behavior of the insects) is probably resulting from the combined modulation of peripheral and central olfactory processes. This hypothesis is further supported by results obtained in response to octenol, where a clear pattern in behavioral response was observed, in spite of a lack of rhythmicity at the periphery. One factor to be considered in this case is that octenol receptors are located in the maxillary palps of *Ae. aegypti* [45,46]. While we found no discernible rhythm in antennal sensitivity, we did observe a significant electrical response to octenol in the EAG recordings that we performed. Therefore, it is possible that *Ae. aegypti* antennal stimulation with octenol at the concentration used (which is high relative to concentrations that mosquitoes would likely encounter in nature) activate nonspecific olfactory receptors (ORs) on the antennae. Interestingly, *Anopheles* mosquitoes also have ORs for octenol located on the maxillary palps [47] while also presenting antennal sensitivity to octenol [48,49]. 

A critical aspect of our experimental design is that it allows us to decouple the rhythm in the behavioral orientation of the insects towards or away from the tested stimuli, from a change of spontaneous locomotor activity levels. Indeed, by taking into account only the mosquitoes that initiate flight and make an active decision between the two test arms of the olfactometer, we ensure that changes in preference indices are not the result of simply having more mosquitoes contributing to the response. In other words, stronger oriented behavioral responses do not mean that more mosquitoes flew, but rather, that among the number of mosquitoes that flew, the responses were more biased towards/away from the odorant stimulus. 

Furthermore, an analysis of the proportion of active mosquitoes (meaning mosquitoes making a choice in the olfactometer) revealed that the presence of odorants acted as an activator of behavior. The proportion of active mosquitoes was indeed close or superior to 50%, even at times where spontaneous activity is otherwise usually observed at minimal levels (e.g., ZT 5–7 and 17–19, Figure 1 and Figure 3). This phenomenon is consistent with what has been previously described (e.g., [50]), but we provide here, to our knowledge, the first demonstration of the time-dependent nature of this olfactory activation of behavior. This highlights the importance of the olfactory context when assessing the epidemiological impact of mosquito circadian rhythms. 

## 5. Conclusions

In the present study we investigated daily olfactory rhythms in adult female *Ae. aegypti* mosquitoes; specifically, we compared patterns in locomotor activity, olfactory sensitivity, and olfactory behavior. In contrast to previous observations in other mosquito species, we observed an odorant-specific modulation of olfactory sensitivity, decoupled from the rhythms in olfactory behavior. Furthermore, we provide, to our knowledge, the first observation of time-dependence in olfactory activation of behavior in *Ae. aegypti* mosquitoes. Altogether, these results suggest that the olfactory behavior of *Aedes* mosquitoes is modulated throughout the day by both peripheral and central processes. This work sets the basis for future work on mosquitoes that would unravel the mechanisms of central modulation and provide strong impetus for identifying the neural and molecular substrates of the modulation of mosquito olfactory behavior, as they could be leveraged as potential targets to disrupt mosquito-host interactions.

## Figures and Tables

**Table 1:** Analysis of variance (ANOVA) reveals that odorant sensitivity of female *Ae. aegypti* mosquitoes is significantly modulated by the odorant stimulus, the time of day, and the interaction between these two factors. **Df**: Degrees of Freedom; **Sum Sq:** sums of squares; **Mean Sq:** mean of squares; **F value:** values of the F statistic; **Pr(<F):** the probability of observing an F ratio greater than the corresponding F value.

	Df	Sum Sq	Mean Sq	F Value	Pr(>F)
Odor	6	1419	236.46	24.711	<2 × 10^−16^
Time of day	3	78	26.05	2.722	0.0437
Odor:Time of day	18	435	24.15	2.524	0.0005
Residuals	557	5330	9.57		
